# Molecular Insights into Ischemia–Reperfusion Injury in Coronary Artery Disease: Mechanisms and Therapeutic Implications: A Comprehensive Review

**DOI:** 10.3390/antiox14020213

**Published:** 2025-02-13

**Authors:** Sai Nikhila Ghanta, Lakshmi. P. V. Kattamuri, Adetayo Odueke, Jawahar L. Mehta

**Affiliations:** 1 Division of Cardiology, University of Arkansas for Medical Sciences, Little Rock, AR 72205, USA; snghanta@uams.edu (S.N.G.); aodueke@uams.edu (A.O.); 2 Department of Internal Medicine, Texas Tech University Health Sciences Center, El Paso, TX 79905, USA; lkattamu@tthsc.edu

**Keywords:** hypoxia, inflammation ischemia–reperfusion injury, oxidative stress, reactive oxygen species

## Abstract

Coronary artery disease remains a leading cause of morbidity and mortality worldwide. Acute myocardial infarction results in ischemia-induced cellular dysfunction and death. While timely reperfusion limits myocardial damage, it paradoxically triggers ischemia–reperfusion injury (IRI), exacerbating tissue damage. IRI, first observed in the 1960s, is mediated by complex molecular pathways, including oxidative stress, calcium dysregulation, endothelial dysfunction, and inflammation. This review examines emerging therapeutic strategies targeting IRI, including ischemic preconditioning, postconditioning, pharmacological agents, and anti-inflammatory therapies. Preconditioning serves as an endogenous protection mechanism, while pharmacological postconditioning has become a more clinically feasible approach to target oxidative stress, inflammation, and apoptosis during reperfusion. Pharmacological agents, such as GSK-3β inhibitors, JNK inhibitors, and mesenchymal stem cell-derived exosomes, have shown promise in modulating molecular pathways, including Wnt/β-catenin and NF-κB, to reduce myocardial injury and enhance recovery. Combination therapies, integrating pharmacological agents with mechanical postconditioning, provide a synergistic approach to further protect tissue and mitigate damage. However, translating preclinical findings to clinical practice remains challenging due to discrepancies between animal models and human conditions, particularly with comorbidities such as diabetes and hypertension. Continued research is essential to refine these therapies, optimize clinical application, and address translational challenges to improve outcomes in IRI.

## 1. Introduction

Coronary artery disease (CAD) is a leading cause of mortality and morbidity, affecting more than 315 million people worldwide [1]. Despite the advancements in increased understanding of its pathophysiology and management, CAD poses a significant financial burden in the United States (US) healthcare system, costing more than USD 600 billion per year [2]. Following ischemia, cardiac tissues suffer cellular dysfunction, eventually leading to cell death, termed acute myocardial infarction (AMI) [3]. Prompt restoration of blood flow is crucial to preserve the at-risk myocardium and minimize infarct size and is the standard treatment to improve cardiovascular outcomes [4]. However, paradoxically, reperfusion itself has been proposed to cause further irreversible tissue damage, “ischemia–reperfusion injury (IRI)”, threatening the viability and function of the myocardium. Myocardial IRI was also postulated in patients undergoing cardioplegic arrest for various cardiovascular/surgeries [5]. The concept of myocardial IRI was first introduced by Jennings et al. in a canine heart model using the coronary ligation technique [6]. They observed that restoration of blood flow after 30–60 min of ischemia accelerated the progression of necrosis, with histopathological changes comparable to the necrosis seen after 24 h of total coronary artery occlusion [6]. This finding raised the question of whether reperfusion itself was a direct cause of tissue injury or it merely expedited the pre-determined process of tissue necrosis. However, the distinction between ischemia and reperfusion became clear only after the discovery of ischemic preconditioning. Murry et al., in 2018, reported that brief periods of ischemia, followed by a prolonged period of ischemia and reperfusion, significantly reduced infarct size in canine heart models [7]. This phenomenon was later demonstrated in various animal models and humans [8]. Ischemic preconditioning is now a widely recognized evolutionarily conserved mechanism, with subsequent experiments highlighting the critical role of the reperfusion event in triggering molecular cascades that can mediate cardioprotection [9].

Despite the numerous advancements made in understanding the pathophysiology and molecular mechanisms underlying ischemia–reperfusion injury and the identification of novel therapeutic targets, many are still in translation to be applied in clinical practice. In addition, numerous molecular mechanisms identified in animal studies remain underexplored in humans, and large-scale clinical trials evaluating promising interventions to minimize IRI in patient care settings are still lacking [5]. We believe the primary reasons for the paucity of human studies and the under-documentation of IRI in clinical settings are not due to its rarity but rather because myocardial IRI is not routinely investigated. Experimental models differ from clinical studies, as they typically involve abrupt occlusion and reperfusion protocols in animals with previously healthy myocardium. In contrast, clinical settings often feature pre-existing progressive CAD, intermittent coronary artery occlusion, and delayed perfusion [7].

In this article, we aim to review the molecular mechanisms, key advancements in basic science in the last decade, and novel therapeutic implications in patients experiencing myocardial ischemia- and reperfusion-associated myocardial injury.

## 2. Pathophysiologic Basis of Ischemia–Reperfusion Injury

The molecular and cellular mechanisms underlying IRI are highly complex, involving the convergence of diverse biological pathways. However, the extent to which these pathways are relevant in humans remains uncertain, as animal models often fail to accurately replicate the IRI in humans. Despite these challenges, several critical pathophysiological processes and molecular signaling pathways of clinically significant IR have been identified (Table 1).

During ischemia, anaerobic glycolysis becomes the primary energy source as oxidative phosphorylation stops, providing just enough adenosine triphosphate (ATP) to meet basic cardiomyocyte energy demands temporarily [10]. However, this process also causes the accumulation of H^+^ ions and intracellular acidosis. Prolonged ischemia eventually depletes ATP reserves, resulting in contracture rigor and cellular damage [10]. Upon reperfusion, mitochondrial oxidative phosphorylation rapidly resumes, but contractile function lags, a phenomenon known as myocardial stunning, characterized by decreased mechanical efficiency and increased oxygen consumption for the same level of work [11]. Paradoxically, the rapid restoration of intracellular pH during reperfusion exacerbates cellular damage: accumulated H^+^ ions are expelled in exchange for Na^+^ via pH-regulatory mechanisms, increasing intracellular Na^+^ levels [12,13]. This activates the 2Na^+^/Ca^2+^ exchanger, leading to an influx of extracellular Ca^2+^ and an increase in sarcoplasmic reticular Ca^2+^, resulting in calcium overload [14,15]. The excess Ca^2+^ triggers hypercontractility; impaired relaxation; mitochondrial damage; and, in severe cases, cell death [16,17].

Cardiac myocytes rely on a continuous supply of ATP to meet their high energy demands, which are primarily generated by mitochondria. Mitochondria also harbor reactive intermediates and proapoptotic signals that make them central to IRI. The metabolic changes associated with ischemia create an environment conducive to the opening of the mitochondrial permeability transition pore (mPTP), a nonselective channel in the inner mitochondrial membrane [18]. The opening of the mPTP—termed “permeability transition”—leads to dissipation of the electrochemical gradient, the release of reactive oxygen species (ROS), and the initiation of apoptosis through apoptosome formation [19,20]. The extent of mPTP opening determines cell fate: minimal opening allows for recovery, moderate opening induces programmed cell death, and severe opening results in necrosis due to energy failure. Thus, the mPTP plays a pivotal role in IR injury, making it a key therapeutic target for mitigating myocardial IRI [21].

ROS are key mediators of myocardial IRI, directly damaging DNA, proteins, and lipids while triggering pro-inflammatory pathways [22]. ROS accumulation also disrupts mitochondrial function by inducing the opening of the mitochondrial permeability transition pore (mPTP), which perpetuates a cycle of “ROS-induced ROS release” [23]. This damage is further compounded by the endothelium’s role in IRI, as excessive production of nitric oxide (NO) under stress conditions reacts with ROS to form peroxynitrite, a highly reactive and toxic molecule [24].

The inflammatory response to ROS involves a cascade of cytokines, including tumor necrosis factor-alpha (TNF-α), which stimulates TNF receptor type 1 on cardiomyocytes, resulting in contractile dysfunction, hypertrophy, fibrosis, and cell death. Additionally, intracellular Ca^2+^ accumulation promotes the formation of calcium pyrophosphate complexes and xanthine oxidoreductases generate uric acid via ROS, which act as danger signals [25]. These signals activate inflammasomes and protein complexes that mediate the production of interleukin-1β (IL-1β) and stimulate Toll-like receptors (TLRs), activating the NF-κB pathway and amplifying the release of additional cytokines and chemokines [26,27].

During myocardial reperfusion, chemoattractants like (TNF-α) and IL-1β rapidly recruit neutrophils into the infarct zone, with their migration into myocardial tissue peaking over the next 24 h [28]. Facilitated by cell-adhesion molecules like intercellular adhesion molecule-1 and E-selectin, these neutrophils cause vascular plugging, release degradative enzymes and generate ROS, exacerbating myocardial damage [28]. This inflammatory response is closely tied to metabolic disruptions, such as hypoxia-induced metabolic acidosis, which compromises tissue integrity and cell survival [13]. Neutrophil-driven inflammation interacts with energy regulation pathways, where IL-6 suppresses myocardial glucose metabolism by inhibiting AMP-activated protein kinase (AMPK), a key energy sensor during ischemia [29]. Since AMPK activation is critical for glucose utilization and energy production in stressed myocardium, targeting inflammation could restore metabolic balance and improve outcomes [30].

During myocardial IRI, autophagy is activated as a protective mechanism via key proteins like the ULK1-Atg13 complex, interacting with mTORC1 [31,32,33]. Processes such as LC3 lipidation and mTOR inhibition (e.g., by rapamycin) promote autophagosome formation, particularly under energy deprivation [31,32]. Despite this, mitochondrial dysfunction, calcium overload, and ROS accumulation during reperfusion can overwhelm autophagy, leading to mPTP formation and cell death [34].

In the catheterization laboratory, the no-reflow phenomenon, seen in 30% of cases, is regarded as one of the most dreaded complications and is, in fact, the most angiographically apparent form of myocardial IRI [35,36]. This is thought to be related to microvascular plugging with leukocytes and platelet debris and plays an important role in myocardial IRI [37,38].

In addition, apoptosis (programmed cell death) plays a significant role in cell death following IR, as demonstrated in rodent models [39]. While prolonged myocardial ischemia primarily leads to necrosis, reperfusion paradoxically accelerates apoptosis by restoring the energy required for its completion [40,41]. Studies in rats show transitions from ischemia-induced necrosis to apoptosis, with apoptosis accounting for up to 86% of cell death after prolonged ischemia [39]. Mitochondrial dysfunction, cytochrome C leakage, and caspase activation following mPTP opening are key mechanisms driving apoptosis, with its extent depending on the duration of reperfusion [42,43]. Contrasting findings in rabbits and dogs suggest that apoptosis is triggered by reperfusion rather than ischemia alone, though species differences and assay variability may influence the results [41,44].

## 3. Molecular Signaling Pathways

In this review, we have specifically included most of the pathways that have been demonstrated to exhibit pathophysiological effects in myocardial IRI based on evidence from animal and experimental models. The selection of these pathways was guided by their established relevance in contributing to the underlying mechanisms of injury and repair in this context.

### 3.1. Wnt Signaling Pathway

The Wnt signaling pathway is a regulator that plays a role in several key cellular processes, such as proliferation, differentiation, migration, and development [45,46]. It can be categorized into two types based on its reliance on β-catenin: canonical and non-canonical pathways. When Wnt ligands are present, the Wnt/β-catenin pathway activates by binding the ligands to Frizzled receptors and co-receptors LRP5/6. This binding triggers the transcription of downstream target genes of the Wnt pathway. Without Wnt ligands, β-catenin is marked for degradation by a destruction complex [47]. On the other hand, the non-canonical Wnt signaling pathway functions independently of β-catenin. It encompasses the Wnt/planar cell polarity (PCP) and Wnt/Ca^2+^ pathways; both triggered when Wnt ligands attach to Frizzled proteins and ROR1/2 [48]. In the Wnt/Ca^2+^ pathway, activated phospholipase C (PLC) generates inositol trisphosphate (IP3), resulting in a significant rise in intracellular Ca^2+^ levels. This activates Ca^2+^-dependent effectors such as calmodulin-dependent protein kinase II (CaMKII), protein kinase C (PKC), calcineurin, and the nuclear factor of activated T cells (NFAT), which commence the transcription of genes linked to Ca^2+^-related function signaling [49]. The Wnt signaling pathway, typically inactive in normal conditions and essential for early heart development, plays a significant role in cardiovascular diseases. In cases of the myocardium, this pathway is involved in multiple IRI-related processes, such as apoptosis, inflammation, oxidative stress, ECM remodeling, angiogenesis, cardiac hypertrophy, and fibrosis [28]. Myocardial IRI suppresses Wnt/β-catenin signaling and increases apoptosis [50]. Non-canonical Wnt/PCP and Wnt/Ca^2+^ pathways are also linked to apoptosis activation. It is proposed that the Wnt/Ca^2+^ pathway leads to Ca^2+^ overload during myocardial I/R, triggering caspase-8 activation, generating free radicals, and producing nitric oxide, collectively driving apoptosis. Consequently, the ryanodine receptors, which are Wnt-associated Ca^2+^ channels, experience various modifications during myocardial IR, such as redox changes and phosphorylation. These alterations prompt the abnormal opening of diastolic ryanodine receptor channels, contributing to ventricular remodeling, arrhythmias, and premature heart failure [51].

**Figure 1:**
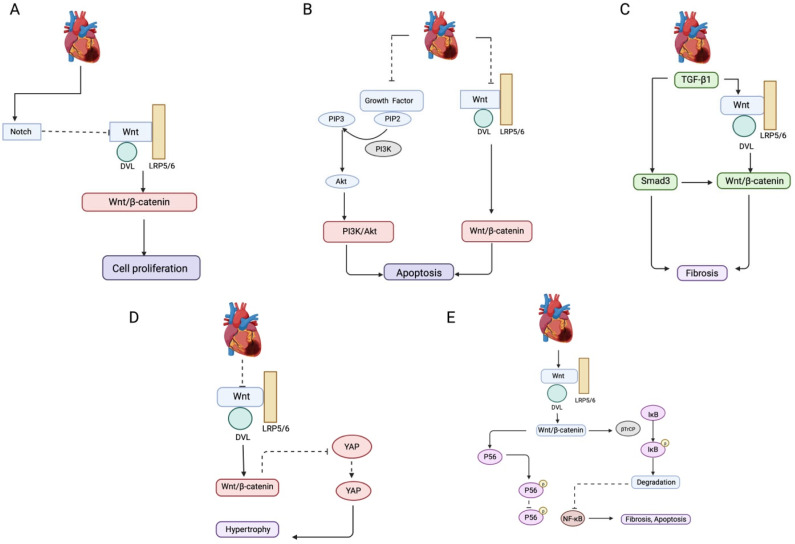
The interplay of Wnt, Notch, PI3K/Akt, TGF-β, NF-κB, and Hippo-YAP signaling pathways during ischemia–reperfusion injury. (**A**) Crosstalk between Wnt and Notch signaling pathway during ischemia–reperfusion injury. Activated Notch signaling inhibits the Wnt signaling transduction and restores the proliferation ability of certain cardiomyocytes. (**B**) Crosstalk between Wnt and PI3K/Akt signaling pathway during ischemia–reperfusion injury. PI3K/Akt and Wnt/β-catenin signaling pathways are downregulated in ischemia–reperfusion injury. This downregulation inhibits the crosstalk between these pathways, ultimately resulting in cardiomyocyte apoptosis and left ventricular dysfunction. (**C**) Crosstalk between the Wnt and TGF-β signaling pathways during ischemia injury. After myocardial ischemia, there is an increased expression of TGF-β1, which activates the Wnt/β-catenin signaling pathway. These two pathways work synergistically to promote the process of myocardial fibrosis. (**D**) Crosstalk between the Wnt and Hippo-YAP signaling pathways during ischemia–reperfusion injury. Following myocardial ischemia–reperfusion injury, the Wnt/β-catenin signaling is downregulated, leading to the inhibition of YAP1 transcription. Consequently, the activity of the Hippo-YAP signaling pathway is suppressed. This cooperative effect between the two signaling pathways contributes to the promotion of myocardial hypertrophy. (**E**) Crosstalk between the Wnt and NF-κB signaling pathways during ischemia–reperfusion injury. The upregulated Wnt/β-catenin signaling pathway facilitates the activation of the NF-κB signaling pathway by promoting nuclear translocation of p65; this activation induces the migration of cardiac fibroblasts. Activated Wnt/β-catenin signaling promotes the degradation of phosphorylated IκB mediated through β-TrCP, subsequently promoting the nuclear translocation of NF-κB, leading to myocardial fibrosis and apoptosis. Wnt: wingless-related integration site; Notch: neurogenic locus notch homolog protein; PI3K: phosphatidylinositol-4,5-bisphosphate 3-kinase; AKT: protein kinase B; TGF-β: transforming growth factor β; NF-Kβ: nuclear factor Kappa β; Hippo-YAP: Hippo-Yes-associated protein; IκB: inhibitor of nuclear factor Kappa β; β-TrCP: β-transducin repeat-containing-protein. Dotted arrows indicate inhibition, and regular arrows indicate activation of the pathways.

A rapid increase in cell death can initiate an inflammatory response and activate cell repair mechanisms. The Wnt signaling pathway has emerged as a key regulator of inflammatory responses in cases of myocardial IRI, particularly in acute MI [48]. After MI, macrophages assume specific roles in the remodeling of the left ventricle. Pro-inflammatory macrophages (M1) are predominantly present in the infarct area during the acute early stages. In contrast, anti-inflammatory macrophages (M2) are primarily recruited into the cardiac tissue microenvironment during the late healing stages [52].Macrophage transformation aids in reducing inflammation and repairing injured myocardium, but persistent activation of the M1 macrophage phenotype worsens the inflammatory response by releasing IFN-γ, which results in cardiomyocyte apoptosis and accelerated myocardial damage [53]. Both canonical and non-canonical Wnt signaling pathways can promote the polarization of macrophages toward the M1 phenotype and inhibit M2 phenotype polarization. Therefore, Wnt signaling activation in cardiomyocytes following ischemia may induce cell death in a macrophage-dependent manner, ultimately aggravating myocardial IRI [54]. By overexpressing Wnt Inhibitory Factor 1 using an adeno-associated virus, the activation of non-canonical Wnt signaling is effectively inhibited, lowering IL-1β and IL-6 levels, thereby producing an anti-inflammatory effect in heart tissue following acute myocardial injury [55]. Wnt/β-catenin signaling inhibition in myocardial IRI also causes intracellular oxidative stress, elevated mitochondrial membrane permeability, and cyt c release, ultimately inducing cardiomyocyte apoptosis and propagating further cardiac damage [56]. These observations suggest that targeting the upstream components of the Wnt signaling pathway, or the path itself, might inhibit apoptosis and reduce myocardial injury in I/R by reversing Wnt signaling. Figure 1 demonstrates the pathophysiological effects of the Wnt signaling pathway in MI.

### 3.2. Crosstalk with Other Pathways

#### 3.2.1. Notch Signaling Pathway

The Notch signaling pathway, crucial for embryonic development and tissue repair, plays a significant role in organ IRI [57,58]. It interacts with the Wnt signaling pathway to regulate processes like cell proliferation, apoptosis, fibrosis, and inflammation [59,60]. The canonical Notch pathway involves five ligands (e.g., jagged1, jagged2, and Delta-like1-4) and four receptors (Notch1-4), where ligand binding activates Notch receptors, releasing intracellular domains that move to the nucleus and induce target gene transcription via the transcription factor cardiolipin synthetic lecithin [61]. The non-canonical Notch pathway, independent of CSL, regulates gene transcription through interactions with other signaling pathways [58]. In myocardial IRI, Notch signaling activation reduces cardiomyocyte death and infarct volume and improves cardiac function [62]. In zebrafish, Notch activation inhibits Wnt signaling, and disrupting Notch in Wnt-inhibited models partially restores cardiomyocyte proliferation, indicating an antagonistic relationship between Notch and Wnt signaling during cardiac repair [63]. 

#### 3.2.2. PI3K/Akt Signaling Pathway

The PI3K/Akt signaling pathway, crucial for regulating cell proliferation, survival, and apoptosis, interacts with the Wnt signaling pathway, influencing IRI in the heart [64]. PI3K activation converts phosphatidylinositol 3,4-bisphosphate into phosphatidylinositol 3,4,5-trisphosphate, a secondary messenger that activates AKT, a kinase that regulates downstream targets like mTOR, NF-κB, and Bad [65]. In myocardial IRI, inhibiting calpain activity increases both PI3K and Wnt/β-catenin signaling, improving blood vessel density and cardiomyocyte survival [66]. In rat cardiomyocytes treated with hypoxia/reoxygenation, Akt1 and Wnt11 overexpression reduced apoptosis, an effect blocked by inhibitors or antibodies against Wnt11 or Akt1 [67].Therapeutics that activate PI3K/Akt/GSK-3β, such as Phyllanthus emblica, reduce myocardial IRI [68].

#### 3.2.3. TGF-β Signaling Pathway

The TGF-β signaling pathway, through Smad-dependent and Smad-independent mechanisms, plays a crucial role in the development of diseases like fibrosis and cancer [69]. It interacts with Wnt signaling by forming TGF-β-stimulated Smad3/β-catenin complexes, promoting β-catenin nuclear translocation and activating Wnt/β-catenin signaling. This crosstalk contributes to processes like apoptosis and fibrosis in myocardial IRI [70]. 

TGF-β binds to its receptors (TβR-I, II, and III), activating receptor-regulated Smads (R-Smads) in the Smad-dependent pathway, which translocate to the nucleus with Co-Smads to regulate gene expression [71]. The Smad-independent pathway activates cascades such as MAPK, PI3K/AKT, and JNK. Together, TGF-β and Wnt/β-catenin signaling synergistically facilitate tissue injury responses, including fibrosis in MI [72].

#### 3.2.4. Hippo-YAP Signaling

The Hippo-YAP signaling pathway interacts with Wnt signaling to regulate myocardial development and IRI [73]. After MIRI, Wnt/β-catenin activation enhances YAP1 transcription, inhibiting Hippo-YAP signaling and reducing hypertrophy. Blocking Hippo-YAP signaling can mitigate fibrosis, promote regeneration, and repair MI damage [73,74]. Additionally, YAP/TAZ influences β-catenin stability in canonical Wnt signaling and may prevent cardiac fibrosis via non-canonical Wnt signaling [75].Targeting the Hippo-YAP and Wnt/β-catenin crosstalk offers potential therapeutic strategies for IRIs [76].

## 4. Therapeutic Strategies for Ischemia–Reperfusion Injury

Many therapeutic strategies focusing on IRI have become vital for improving patient outcomes, considering the major role in the fatality rate associated with cardiovascular diseases. These strategies include a combination of preconditioning, postconditioning, pharmacological agents, and anti-inflammatory therapies that are majorly focused on reducing the pathological effects of myocardial IRI. 

### 4.1. Preconditioning and Postconditioning Approaches

Preconditioning is an inbuilt mechanism of protection against ischemic damage. It involves exposing cells to short periods of ischemia, leading to the activation of protective pathways to make the cells more resistant to subsequent, long-term insults. However, its clinical application is limited due to its reliance on the prediction of ischemic events [77]. On the other hand, postconditioning, especially pharmacological postconditioning (PPC), has become a flexible and clinically more viable applicable technique. PPC includes administering medications at the start of reperfusion to reduce and avoid I/R-induced damage via several molecular pathways [78]. Wu et al. have proven the beneficial role of PPC in safeguarding the myocardium by improving oxidative stress, inflammation, calcium overload, and apoptosis. This method has significant potential as it can be used during reperfusion without any need for invasive procedures. This makes it more practical for clinical use at a large level. Moreover, the PPC can be adjusted for different conditions, which makes it a flexible and effective method for limiting myocardial infarct size and improving cardiac functions after ischemia [78].

### 4.2. Pharmacological Agents

For reducing the IRI, pharmacological agents that modulate specific signaling pathways have shown significant results. Among these, the most significant ones and of particular interest are the glycogen synthase kinase-3β (GSK-3β) inhibitors, c-Jun N-terminal kinase (JNK) inhibitors, and exosomes from mesenchymal stem cells because of their ability to affect key molecular mechanisms included in myocardial IRI.

#### 4.2.1. Xanthine Oxidase Inhibitors

Xanthine oxidase inhibitors, such as allopurinol and oxypurinol, reduce ROS production but have inconsistent efficacy, often requiring high doses. These inhibitors also reduce xanthine oxidase’s nitrate reductase activity, thereby lowering nitric oxide production and diminishing its vasodilatory benefits during IRI. The Nox/Duox family of NADPH oxidases also contributes to ROS production during IRI, generating superoxide and hydrogen peroxide in response to activators such as HIF-1α, phospholipase A2, TNF-α, IL-1β, interferon-γ, and angiotensin II [79]. Superoxide permeates cell membranes via ion channels, while hydrogen peroxide enters the cytoplasm, leading to cellular damage. Pharmacological inhibitors of Nox-derived ROS, including apocynin, diphenyleneiodonium, and curcumin, show promise in reducing oxidative stress in cardiac, lung, and brain cells, but their therapeutic potential in IRI remains under investigation [80].

#### 4.2.2. Trimetazidine

Trimetazidine exemplifies the benefits of metabolic modulation, as it inhibits long-chain 3-ketoacyl-CoA thiolase, shifting energy reliance from fatty acid oxidation to glucose metabolism. This shift reduces inflammation and ischemia–reperfusion damage, with clinical evidence demonstrating its protective effects on cardiac function and survival [81]. A meta-analysis of randomized controlled trials in heart failure demonstrated that trimetazidine significantly reduced all-cause mortality, cardiovascular events, and hospitalizations [82].

#### 4.2.3. GSK-3β Inhibitors

GSK-3β inhibition has resulted in reduced myocardial remodeling and promoted angiogenesis, especially after acute MI. For example, Padrón-Barthe et al. (2019) demonstrated that the allosteric inhibitor NP12 had beneficial results in reducing myocardial infarct size by stabilizing β-catenin in the Wnt/β-catenin signaling pathway [83]. NP12 decreases apoptosis and fibrosis while enhancing angiogenesis by inhibiting GSK-3β. Therefore, it improves myocardial function and increases survival chances in the aftermath of AMI [84]. NP12 has been very effective in increasing tissue repair and reducing fibrosis, which are important factors in the recovery of heart function following IRI. In a study by Baruah et al., NP12 administration increased the expression of vascular endothelial growth factor receptor 2 (VEGFR2) and β-catenin. This promoted vascular proliferation and repair in the damaged myocardium [84].

#### 4.2.4. JNK Inhibitors

JNK inhibitors, like SP600125, target non-canonical Wnt signaling pathways and result in a reduction in IRI-induced apoptosis and inflammation by modulating the pro-inflammatory cytokines and apoptotic protein production [48]. In a study by Lojk and Marc et al., the JNK inhibitor SP600125 attenuated non-canonical Wnt signaling, lowering the activation of pathways that cause cell death and inflammation [85]

### 4.3. Anti-Inflammatory Therapies

#### 4.3.1. NF-κB Inhibitors

NF-κB is an important transcription factor that is included in the inflammatory response during IRI. In the study by Dong et al., NF-κB inhibitors like dexamethasone and other pharmacological agents attenuate the inflammatory cascade activated during reperfusion, thereby reducing myocardial damage [86].

#### 4.3.2. RAGE Inhibitors

Another critical mediator of inflammation in IRI is the receptor for advanced glycation end products (RAGE), whose activation releases pro-inflammatory cytokines and contributes to endothelial dysfunction and tissue [87]. RAGE inhibitors like soluble RAGE or specific monoclonal antibodies have been discovered to block the harmful effects of RAGE signaling in the experimental models of myocardial IRI. These inhibitors also help to reduce the expression of inflammatory cytokines and improve tissue function [87].

### 4.4. Emerging Therapies

The field of regenerative medicine has seen remarkable advancements in recent years, with novel therapies showing potential to address tissue defects and injuries. Among these, exosome-based therapy and combination therapies are at the forefront of developing effective treatment strategies.

#### Exosome-Based Therapy

Exosomes, small membrane-bound vesicles secreted by various types of cells like mesenchymal stem cells, have gained much interest because of their ability to mediate cell-to-cell communication as well as promote tissue repair. In the context of IRI, exosomes coming from adipose-derived mesenchymal stem cells (ADMSCs-ex) play a significant role in myocardial protection by activating the Wnt/β-catenin signaling pathway [88]. ADMSCs-ex have been shown to decrease the infarct size, relieve apoptosis, and improve myocardial function. This is achieved by enhancing β-catenin expression and stabilizing key components of the Wnt signaling cascade. Cui et al. (2017) also found that administering ADMSCs-ex to rats with myocardial IRI notably reduced myocardial infarction, and it also improved cell viability by modulating apoptotic pathways [88].

Exosome-based therapy, especially using MSC-derived exosomes, has emerged as a promising approach for tissue regeneration, offering several advantages over traditional stem cell-based treatments. These extracellular vesicles carry a cargo of bioactive molecules including proteins, lipids, mRNAs, and miRNAs that help intercellular communication and also modulate various biological processes such as inflammation, cell migration, and tissue repair [89].

MSC-derived exosomes replicate the biological effects of their parental MSCs by transferring their cargo to recipient cells in the local microenvironment, which can promote tissue regeneration by activating critical signaling pathways, and it also includes the Wnt/β-catenin pathway. For example, the exosomes derived from adipose mesenchymal stem cells (ADSC-Exosomes) accelerate cutaneous wound healing by activating this pathway, which in turn promotes cell proliferation and migration and inhibits apoptosis [90]. Li et al. (2024) studied that the exosomes from human umbilical mesenchymal stem cells (hucMSCs) enhance wound healing by upregulating proteins related to cell migration and collagen deposition [91].

One of the significant advantages of MSC-derived exosomes over whole-cell therapies is their non-immunogenic nature and ease of use. Unlike MSCs, exosomes do not pose the risk of tumorigenesis or immune rejection, making them a safer alternative for clinical applications [91]. Furthermore, exosomes are highly stable under various conditions, making them easier to store and deliver, thus facilitating their large-scale production and clinical implementation [89]. While MSC-derived exosomes hold considerable promise in regenerative medicine, challenges remain in optimizing their production, improving their therapeutic efficacy, and understanding the precise mechanisms underlying their effects. Preconditioning MSCs with various agents to enhance exosome production and their cargo content may provide further improvements [91].

### 4.5. Combination Therapies: Combining Pharmacological Agents with Mechanical Postconditioning

Combination therapies combine pharmacological agents with mechanical postconditioning and provide a synergistic approach that enhances tissue repair and mitigates injury. Mechanical postconditioning (PoCo) includes brief cycles of ischemia and reperfusion, which follows a major ischemic event that has resulted in reduced infarct size and has protected tissues from reperfusion injury [92]. This approach is very beneficial in clinical settings where ischemia is unpredictable such as MI. Vinten-Johansen and Shi et al. (2011) determined that pharmacological agents combined with mechanical postconditioning can enhance its protective effects, like the use of pharmacological postconditioning (pPoCo), which includes adding specific agents during the reperfusion phase, which can decrease the tissue damage by modulating molecular pathways involved in inflammation, apoptosis, and cell survival [93]. Khan et al. identified that agents targeting the PI3K/Akt and NF-κB pathways have shown promise in enhancing the benefits of postconditioning, reducing myocardial injury, and promoting tissue repair in animal models [92]. In clinical practice, the combination of pharmacological agents with mechanical postconditioning could lead to better outcomes by providing a multi-target approach that addresses various aspects of ischemic injury. For instance, in the setting of MI, where reperfusion injury can worsen tissue damage, the integration of postconditioning with agents that promote angiogenesis and inhibit inflammatory responses could improve recovery [9]. 

## 5. Discrepancies Between Preclinical and Clinical Studies

Even though preclinical studies have successfully demonstrated the therapeutic potential of various agents, most clinical trials have failed to replicate these results. This discrepancy highlights the challenges of translating preclinical findings from animal models into effective clinical treatments.

A major challenge in translating preclinical treatments into clinical practice is the difference between animal models and human patients, particularly in terms of underlying health conditions and comorbidities. Preclinical trials often use healthy animals that do not reflect the diversity and complexity of human patients, who frequently suffer from conditions like diabetes, hypertension, and atherosclerosis [43].

For example, exosome-based therapies have shown promising results in preclinical models for wound healing, bone regeneration, and cartilage repair. However, when translated into clinical trials, the efficacy of these therapies has been reduced in patients with diabetes, where chronic inflammation and impaired wound healing are prevalent [92]. Similarly, MSC-based therapies for MI have demonstrated positive results in animal models, but in clinical settings, comorbid conditions such as obesity and coronary artery disease altered the efficacy of the treatment [94].

Moreover, preclinical trials often involve young, healthy animals, which do not account for factors like aging or other chronic conditions that significantly impact the outcomes in human patients. For example, the ischemic preconditioning and postconditioning strategies that are proven in animal models often do not give the same outcomes when used in elderly patients or patients with multiple comorbidities (diabetes, hypertension, etc.). As studied by Hausenloy et al., these factors change the molecular pathways involved in tissue injury and repair, making it difficult to translate preclinical results to clinical settings [43].

The lack of appropriate animal models that accurately replicates human disease conditions, particularly the complex interplay of multiple comorbidities, is a critical issue that hinders the successful translation of preclinical findings into clinical therapies. Future research should focus on improving the design of animal models that better reflect human pathophysiology, including age-related changes and the presence of comorbid conditions, to enhance the predictive value of preclinical studies.

## 6. Conclusions and Future Directions

The present review provides an overview of the pathophysiology of myocardial IRI, emphasizing the role of ROS in triggering cell death; disruptions in the electron transport chain within cardiomyocyte mitochondria; and mitochondrial dysfunction caused by oxidative stress, reduced ATP production, and electrolyte imbalances during reperfusion. Elevated ROS levels contribute to cell damage through autophagy, apoptosis, inflammation, necrosis, and other programmed cell death processes. Despite significant progress, many aspects of myocardial IRI remain incompletely understood, particularly the roles of pyroptosis, ferroptosis, necrosis, and downstream mediators of key signaling pathways. Clinical strategies to limit infarct size and alleviate myocardial IRI include ischemic postconditioning; remote ischemic conditioning; and pharmacological interventions such as cyclosporine, insulin, glucagon-like peptide-1 agonists, β-blockers, and antioxidant therapies like propofol and Salvia miltiorrhiza [95,96,97]. Notably, ischemic preconditioning has demonstrated cardioprotective effects by reducing mPTP opening [98]. The intricate interplay of signaling pathways, including Wnt, Notch, PI3K/Akt, TGF-β, and Hippo-YAP, significantly influences the pathophysiology of IRI and MI. Emerging therapeutic strategies, such as pathway-specific inhibitors, exosome-based therapies, and regenerative medicine approaches, show promise in mitigating myocardial injury and improving outcomes. Further research is essential to refine these therapies, address translational challenges, and enhance their clinical applicability.

## Figures and Tables

**Table 1:** Key pathophysiological processes and molecular signaling pathways implicated in clinically significant ischemia–reperfusion injury.

Pathophysiological processes Intracellular calcium accumulation Intracellular sodium accumulation Rapid pH change with ischemia and reperfusion Loss of mitochondrial membrane potential Oxidative stress Free radical formation/reactive oxygen species (ROS) Uric acid generation ROS-induced ROS generation NO metabolism Endothelial dysfunction Cytokines and chemokine signaling Expression of cell adhesion molecules Neutrophil infiltration Immune activation Platelet aggregation and microembolization Autophagy Apoptosis
Molecular signaling pathways Wnt pathways Notch signaling pathway PI3K/Akt signaling pathway TGF-β signaling pathway Hippo-YAP signaling
